# Increased BOLD Signals Elicited by High Gamma Auditory Stimulation of the Left Auditory Cortex in Acute State Schizophrenia

**DOI:** 10.1016/j.ebiom.2016.09.008

**Published:** 2016-09-13

**Authors:** Hironori Kuga, Toshiaki Onitsuka, Yoji Hirano, Itta Nakamura, Naoya Oribe, Hiroaki Mizuhara, Ryota Kanai, Shigenobu Kanba, Takefumi Ueno

**Affiliations:** a Department of Neuropsychiatry, Graduate School of Medical Sciences, Kyushu University, 3-1-1 Maidashi, Higashiku, Fukuoka 812-8582, Japan; b Division of Clinical Research, National Hospital Organization, Hizen Psychiatric Center, 160 Mitsu, Yoshinogari-cho, Kanzaki-gun, Saga 842-0192, Japan; c Graduate School of Informatics, Kyoto University, 36-1 Yoshida-Honmachi, Sakyo-ku, Kyoto 606-8501, Japan; d Araya Brain Imaging, 1-6-15-301, Hirakawa-cho, Chiyoda-ku, Tokyo 102-0093, Japan

**Keywords:** Acute episode schizophrenia, Bold, High gamma oscillation, Auditory steady state response, Neural over activation, Auditory hallucination

## Abstract

Recent MRI studies have shown that schizophrenia is characterized by reductions in brain gray matter, which progress in the acute state of the disease. Cortical circuitry abnormalities in gamma oscillations, such as deficits in the auditory steady state response (ASSR) to gamma frequency (> 30-Hz) stimulation, have also been reported in schizophrenia patients. In the current study, we investigated neural responses during click stimulation by BOLD signals.

We acquired BOLD responses elicited by click trains of 20, 30, 40 and 80-Hz frequencies from 15 patients with acute episode schizophrenia (AESZ), 14 symptom-severity-matched patients with non-acute episode schizophrenia (NASZ), and 24 healthy controls (HC), assessed via a standard general linear-model-based analysis. The AESZ group showed significantly increased ASSR-BOLD signals to 80-Hz stimuli in the left auditory cortex compared with the HC and NASZ groups. In addition, enhanced 80-Hz ASSR-BOLD signals were associated with more severe auditory hallucination experiences in AESZ participants. The present results indicate that neural over activation occurs during 80-Hz auditory stimulation of the left auditory cortex in individuals with acute state schizophrenia. Given the possible association between abnormal gamma activity and increased glutamate levels, our data may reflect glutamate toxicity in the auditory cortex in the acute state of schizophrenia, which might lead to progressive changes in the left transverse temporal gyrus.

## Highlights

• We investigated neural responses during click stimulation by BOLD signals.
• We evaluated patients with acute and non-acute schizophrenia, and healthy controls.
• 80-Hz auditory stimulation activated the left auditory cortex in acute schizophrenia.
• Our data may reflect left auditory cortex glutamate toxicity in acute schizophrenia.

Recent MRI studies show that schizophrenia is characterized by reduced brain gray matter, which deteriorates in the acute state of the disease. Periodic auditory click trains elicit auditory steady-state responses (ASSRs), and ASSR abnormalities are reported in schizophrenia. We investigated neural responses during click stimulation using BOLD signals, which may reflect glutamate toxicity. Compared with non-acute schizophrenia patients and healthy controls, acute episode schizophrenia patients showed significantly increased ASSR-BOLD to 80-Hz stimuli in the left auditory cortex. Our data demonstrate neuronal over activation in terms of the BOLD pattern in acute state schizophrenia, which might reflect progressive volume reduction in the left superior temporal cortex by glutamate toxicity during the acute phase of schizophrenia.

## 1 Introduction

In a recent Global Burden of Disease Study, ‘acute’ schizophrenia received the highest disability weight out of 220 health state valuations (0–76, where 0 equals no disability and 1 equals complete disability) (Salomon et al., 2012). Recent meta-analyses using magnetic resonance imaging (MRI) show that schizophrenia is characterized by reductions in gray matter, which occur before full symptom onset and progress more quickly in the acute exacerbation state (Olabi et al., 2011) and treatment-resistant state, compared with the treatment-responsive state (Mouchlianitis et al., 2016). For example, Takahashi et al. (2010) reported that first-episode schizophrenia and schizotypal patients showed significant decreases in gray matter volume in the left transverse temporal gyrus and left planum temporale approximately 2–7 years after an initial assessment, compared with control samples. Although the precise neurobiological mechanisms underlying progressive deterioration in the left transverse temporal gyrus and left planum temporale in schizophrenia are unknown, a growing body of work has implicated abnormal excitatory amino acid neurotransmission, possibly mediated by a deficit in recurrent inhibition (Coyle and Konopaske, 2016). Although controversial, this mechanism could elicit ongoing, use-dependent cellular damage mediated via excitotoxic effects. In in vivo studies, increased glutamate levels appear to feature early in the course of illness, rather than during chronic schizophrenia (Marsman et al., 2013). However, glutamate production and toxicity may play a role in the acute state of schizophrenia.

Schizophrenia is also characterized by abnormalities in the cortical circuitry underlying gamma oscillations elicited by a variety of stimuli and tasks (Uhlhaas et al., 2010). Especially, individuals with schizophrenia exhibit deficits in the auditory steady state response (ASSR) induced by gamma frequency (> 30-Hz) stimulation. In healthy individuals, the ASSR contains resonant frequencies around 40-Hz and 80-Hz, with a larger power at 40-Hz (Sivarao, 2015). The power of the ASSR is enhanced at these frequencies. At the cellular level, the generation and maintenance of gamma oscillations critically depend on networks of fast-spiking parvalbumin-expressing gamma aminobutyric acid (GABA)-ergic interneurons (Owen et al., 2016). In addition, *N*-methyl-d-aspartate receptor (NMDAR) signaling in parvalbumin-expressing GABAergic interneurons is critical for the regulation of spontaneous (non-stimulus locked) and evoked gamma oscillations (Carlen et al., 2012). ASSR deficits in individuals with chronic schizophrenia have been correlated with reduced evoked gamma responses (Tsuchimoto et al., 2011) and increased spontaneous gamma activities (Hirano et al., 2015) during auditory stimulation, indicating that neural circuitry abnormalities in schizophrenia patients may be associated with an imbalance between excitatory glutamate and inhibitory GABA neurotransmission. Thus, it is important to investigate both evoked gamma responses and spontaneous gamma activities during auditory stimulation in this population.

**Fig. 1:**
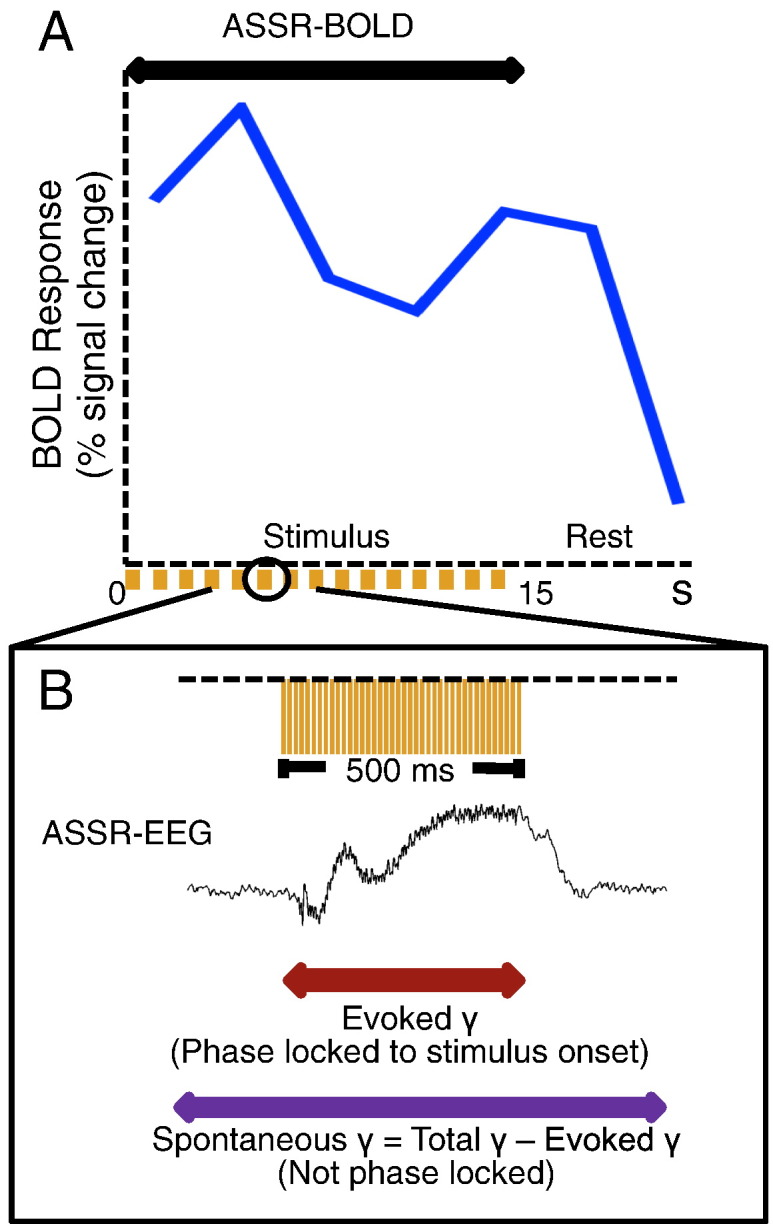
Relationships between fMRI signals and electrophysiological responses for auditory steady state response (ASSR). A. BOLD percent signal change of the left auditory cortex to the 80-Hz ASSR (blue) in healthy controls. Arrows indicate ASSR-BOLD in ASSR stimuli. During 15 s of stimulation, 15 click trains are presented as indicated at the bottom. B. Electroencephalographic response to 80-Hz click stimulation obtained from one healthy subject in an independent session. Arrows indicate evoked γ (red) and spontaneous γ (purple) in EEG. Total power includes both spontaneous γ activity (not phase locked to stimulus onset) and evoked γ activity (phase locked to stimulus onset). The 80-Hz ASSR-stimuli indicated at the top are 1-ms clicks. The duration of the click trains and the inter-train interval was 500 ms.

Although most ASSR studies have used electroencephalography (EEG) and megnetoencephalography (MEG), hemodynamic signals have been found to strongly correlate with synchronized gamma oscillations (Niessing et al., 2005). Niessing et al. reported that hemodynamic responses were significantly and positively correlated with neuronal synchronization in the gamma range (52–90 Hz) in the visual cortex of cats. More recently, specific gamma-BOLD correlations have been reported in humans during a cognitive visual attention task (Scheeringa et al., 2011). Other reports (Logothetis et al., 2001, Logothetis et al., 2010, Kayser et al., 2004, Goense and Logothetis, 2008, Murayama et al., 2010, Scholvinck et al., 2010) have consistently demonstrated the significant involvement of gamma oscillations in neurovascular coupling. In addition, Kann et al. (2011) reported that gamma band neural oscillations were particularly associated with higher mitochondrial oxidative metabolism, which is characterized by higher oxygen consumption and mitochondrial gene expression, indicating significant associations between gamma oscillations and BOLD signals. Therefore, we consider functional MRI (fMRI) to be suitable for evaluating evoked gamma and spontaneous gamma oscillations during periodic click stimuli by blood oxygenation level dependent (BOLD) signals (Fig. 1 shows the relationships between fMRI signals and electrophysiological responses for ASSR).

In this study, we examined beta (ASSR to 20-Hz click trains), low gamma (ASSR to 30 and 40-Hz click trains), and high gamma (ASSR to 80-Hz click trains) ASSR-BOLD signals in healthy controls (HC), patients with acute episode schizophrenia (AESZ), and patients with non-acute phase schizophrenia (NASZ). We sought to clarify whether ASSR-BOLD signals would be altered in patients with schizophrenia, and we compared the AESZ and NASZ groups. Given the potential for increased spontaneous gamma activities during auditory stimulation in the acute state, we hypothesized that BOLD signals elicited by the higher frequency periodic click stimuli would be elevated in the AESZ but not the NASZ group.

## 2 Materials & Methods

### 2.1 Participants

Demographic and clinical data are shown in Table 1. The sample consisted of 24 HC, 15 AESZ, and 14 symptom-severity-matched NASZ subjects. All subjects had normal hearing, were between 25 and 59 years of age, and were right-handed (Oldfield, 1971). After receiving a complete description of the study, all participants signed an informed consent form according to the regulations of the Ethics Committee of the National Hospital Organization Hizen Psychiatric Center. Healthy controls were screened using the Structured Clinical Interview (SCID)-non-patient edition (First et al., 2002a, First et al., 2002b). No healthy controls or their first-degree relatives had an Axis-I psychiatric disorder. The exclusion criteria were: 1) neurological illness or major head trauma, 2) previous treatment with electroconvulsive therapy, 3) alcohol or drug dependence, 4) alcohol or drug abuse within the past 5 years, and 5) a verbal intelligence quotient below 75.

**Table 1:** Demographic and clinical characteristics of the study groups.

	HC	NASZ	AESZ	χ2, F or t	df	*p*-Value
Male/female, n	12/12	7/7	6/9	0.43	2	0.80
Age (years)	38.8 ± 11.8	44.1 ± 9.2	36.9 ± 10.1	1.76	2,50	0.18
Handedness	96.3 ± 9.5	97.5 ± 5.1	86.23 ± 25.3	2.63	2,50	0.08
SES	1.8 ± 0.8	3.5 ± 0.8	2.9 ± 0.9	21.33	2,50	< 0.001
Onset age (years)		29.3 ± 12.1	24.1 ± 5.1	1.53	27	0.14
Duration of illness (months)		175.9 ± 2.5	146.0 ± 114.9	0.76	27	0.45
PANSS-total		42.6 ± 12.9	41.3 ± 15.3	0.36	27	0.73
Positive		23.1 ± 8.1	22.6 ± 8.4	0.15	27	0.88
Negative		18.4 ± 5.7	17.4 ± 6.4	0.46	27	0.65
BPRS-total		65.0 ± 18.5	59.1 ± 19.3	0.84	27	0.41
Chlorpromazine equivalents (mg)		416.8 ± 355.5	615.0 ± 243.1	− 1.76	27	0.09

Values are mean ± SD unless otherwise noted. HC = healthy controls; NASZ = non-acute phase schizophrenia; AESZ = acute episode schizophrenia; SES = socioeconomic status; PANSS = positive and negative syndrome scale; BPRS = brief psychiatric rating scale.

Patients were medicated with antipsychotics (only atypical: 8 NASZ, 9 AESZ; only typical: 1 NASZ, 0 AESZ; both atypical and typical: 4 NASZ, 5 AESZ), and unmedicated with antipsychotics (1 NASZ, 1 AESZ). Within each group, the following numbers of subjects received additional medications: mood stabilizers: 3 NASZ, 2AESZ; anti-anxiolytic drugs: 10 NASZ, 7 AESZ.

All patients were recruited from National Hospital Organization Hizen Psychiatric Center and were diagnosed based on the SCID-DSM IV-TR, Research Version (First et al., 2002a, First et al., 2002b) and information from patient medical records. Based on previous studies, we operationally defined an acute exacerbation episode as that occurring within 4 weeks of psychiatric hospitalization, and a non-acute phase as that occurring at least 4 weeks after psychiatric hospitalization (Hatta and Ito, 2014, Mitra et al., 2015, Li et al., 2016). All AESZ and 11 NASZ participants were inpatients, and 3 NASZ participants were outpatients. The patients were assessed using the Positive and Negative Syndrome Scale (PANSS) (Kay et al., 1987) and the Brief Psychiatric Rating Scale (BPRS) (Bell et al., 1992).

### 2.2 Stimuli and Procedure

Following a hearing test, participants were asked to lay supine inside an MRI scanner while wearing head-phones. The head of each participant was restrained via padding behind the neck and between the head and the coil. Participants were asked to keep their head still inside the scanner and to focus on a fixation cross presented on a screen. All auditory stimuli were delivered binaurally through the head-phones.

We presented auditory stimulation using a 4-min-block-design paradigm with 8 blocks of 15 s of rest (stimulation OFF) and 15 s of stimulation including 15 click trains (stimulation ON). In total, we presented 120 click trains for each fMRI session. We conducted four fMRI sessions with different sound stimuli for each subject. The stimuli were 1-ms clicks, presented binaurally as trains of clicks for each frequency (20, 30, 40, and 80-Hz). Both the duration of the click trains and the inter-train interval were 500 ms, and the click trains were presented with an intensity of 80-dB sound pressure level. The order of sessions was counterbalanced across subjects.

### 2.3 Data Acquisition

We conducted MRI using a 1.5-T Philips scanner with a standard head coil, located at the National Hospital Organization Hizen Psychiatric Center. We used standard sequence parameters to obtain functional images, as follows: gradient-echo echo-planar imaging (EPI); repetition time (TR) = 3000 ms; echo time (TE) = 45 ms; flip angle = 90°; field of view (FOV) = 230 × 230 mm; matrix = 64 × 64; 60 axial slices with a slice thickness of 4 mm with no slice gap. We acquired a high-resolution T1-weighted 3D anatomical image for each participant between the functional data trials.

### 2.4 Image Processing

Raw image DICOM files were converted to the NIFTI format using MRIConvert (Version 2.0, Lewis Center for Neuroimaging, Oregon). Image processing and statistical analyses were performed using the statistical parametric mapping software SPM8 (Wellcome Department of Cognitive Neurology, London, United Kingdom) with Matlab R2014a (The Math Works Inc., Natick, MA). The first five volumes of each EPI image run were excluded to allow the MR signal to reach a state of equilibrium. All volumes of functional EPI images were realigned to the first volume of each session to correct for subject motion, and the mean functional EPI image was then spatially co-registered with the anatomical T1 images. Each co-registered T1-weighted anatomical image was normalized into a standard T1 template image (ICBM 152), which defined the Montreal Neurological Institute (MNI) space. The parameters from this normalization process were then applied to each functional image. The normalized functional images were smoothed with a 3D 8-mm full-width half-maximum (FWHM) Gaussian Kernel. Time series data at each voxel were temporally filtered using a high pass filter with a cutoff of 128 s.

### 2.5 Statistical Analysis

We used one-way analyses of variance (ANOVA), chi-squared tests, and t-tests to assess group differences in the demographic variables.

We performed fMRI statistical analysis on the preprocessed EPIs with the general linear model (GLM) using a two-level approach (Friston et al., 1994). The model consisted of boxcar functions convolved with the canonical hemodynamic response function, and then used as the regressors for the regression analysis. The six head motion parameters, derived from the realignment processing, were also used as regressors to reduce the motion related artifacts. On the first level of analysis, individual contrast images for each stimulus versus rest were computed and taken to the second level for random-effects inference. On the second level, contrast images for stimuli as the within-subject factors were submitted to three groups (HC, NASZ, AESZ) as the between-subject factors full-factorial ANOVA. All fMRI results are reported at a significance level of *p* < 0.05, family-wise error (FWE)-corrected (voxel-level corrected), or *p* < 0.05, FWE cluster-corrected across the whole brain with the initial voxel threshold at *p* < 0.001, uncorrected. To determine the direction of the frequency-by-group interaction, we extracted contrast values by identifying the primary auditory cortex and Brodmann areas 41 and 42 as regions of interest (ROI)s using MarsBar (http://marsbar.sourceforge.net). We chose these areas because the ASSR is reportedly evoked in or near the primary auditory cortex (Herdman et al., 2003, Ross et al., 2005). The resulting contrast values were subjected to ANOVA in SPSS with the three groups (HC, NASZ, AESZ) for each ROI, and we used Bonferroni post-hoc tests to test the differences between the groups.

To visualize the time-course of the responses in each ROI, we fitted a finite impulse response (FIR) model (Dale, 1999, Ollinger et al., 2001) in MarsBar to the data. This involved using a linear model to provide unbiased estimates of the average signal intensity at each time point without making a priori assumptions about the shape of the hemodynamic response (HDR). We modeled seven time-windows of every 3 s (corresponding to the TR), time-locked to the onset of the first auditory stimulation. The average signal used in this calculation is based on frequency and is identified as the value of percent signal change for the mean column of the SPM regression analysis. We performed a repeated-measures ANOVA with time window (0–3, 3–6, 6–9, 9–12, 12–15, 15–18, and 18–21 s) as a within-subjects factor and group (HC, NASZ, AESZ) as a between-subjects factor. The resulting percent signal change values were subjected to a three group (HC, NASZ, AESZ) ANOVA in SPSS, and we used Bonferroni post-hoc tests to assess the differences between groups. We applied the Greenhouse–Geisser correction for inhomogeneity of variance for factors with > 2 levels, as reflected in the reported *p* values.

For the period with the largest main effect of group during auditory stimulation, we performed correlational analyses between hallucination scores in the PANSS (all AESZ patients had auditory hallucinations) and BOLD percent signal changes. Effect sizes are expressed as Cohen's d. The Spearman's rho was used for correlational analysis. For all statistical tests, α was 0.05.

## 3 Results

### 3.1 Demographics

We found no significant group differences in the demographic data except in terms of SES, consistent with reduced functioning due to schizophrenia (Table 1).

### 3.2 Mean ASSR-BOLD

#### 3.2.1 Main Effect of Group and Stimulation

**Fig. 2:**
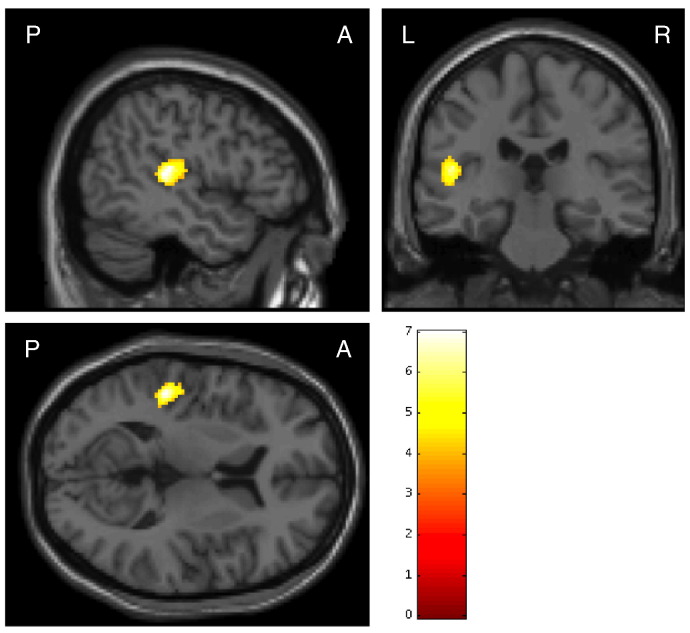
Regions where significant group-by-frequency interactions were revealed by fMRI analyses. Colored bars represent the F-values of the interactions (cluster-level FWE corrected *p* < 0.05).

Our full-factorial ANOVA revealed no significant group differences. The main effect of frequency was associated with significant bilateral activity in Brodmann areas 41 and 42 (left: − 54, − 26, 6, cluster size = 656, F[3200] = 15.38, FWE corrected *p* < 0.001; right: 58, − 20, 4, cluster size = 297, F[3200] = 9.32, FWE corrected *p* = 0.003), indicating that the 80-Hz stimulation evoked significantly larger BOLD responses, compared with the other stimulation frequencies. Furthermore, we found significant group-by-frequency interactions in the left transverse temporal gyrus, including Brodmann areas 41 and 42 (cluster level FWE corrected *p* = 0·001), as shown in Fig. 2 and Table 2. Thus, we focused our remaining analyses on the ASSR-BOLD in Brodmann areas 41 and 42.

**Table 2:** The fMRI result of anatomical regions, seed voxel coordinates (MNI), and F-value for the significant group-by-frequency interactions.

Cluster size (mm^3^)	MNI coordinate (mm)			F-value	Anatomical region
	x	y	z		
2040	− 48	− 30	10	7.00	Left transverse temporal gyrus/left planum temporale
	− 48	− 28	14	6.93	Left parietal operculum

Results are thresholded at *p* < 0.05, cluster-corrected FWE. MNI: Montreal Neurological Institute.

#### 3.2.2 ASSR-BOLD Contrast in Brodmann Areas 41 and 42

**Fig. 3:**
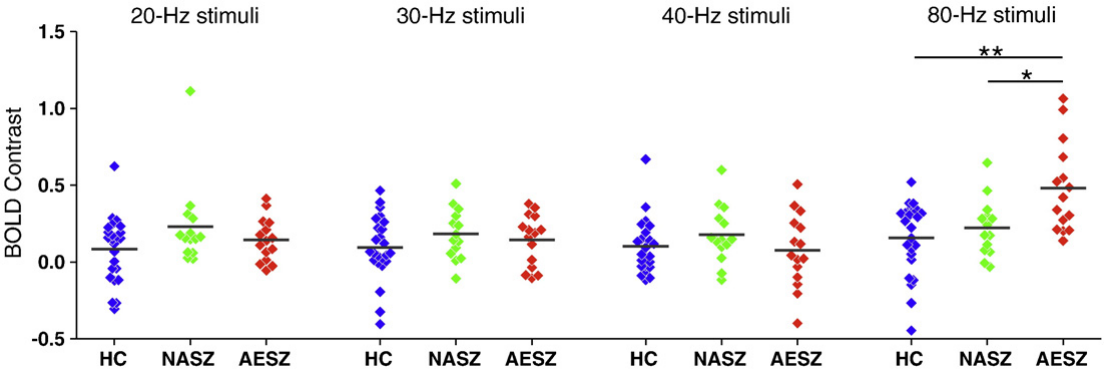
Scattergrams of the BOLD contrast of 20-Hz, 30-Hz, 40-Hz, and 80-Hz ASSR stimulation between healthy controls [(HC); blue squares], non-acute phase schizophrenia patients [(NASZ); green squares], and acute episode schizophrenia patients [(AESZ); red squares]. The AESZ group showed significantly increased ASSR-BOLD signals to the 80-Hz stimuli compared with the NASZ (*p = 0.018, d = 1.08) and HC groups (**p = 0.001, d = 1.28). The BOLD contrast refers to the beta-weight of the event of interest. Horizontal lines indicate group means.

**Fig. 4:**
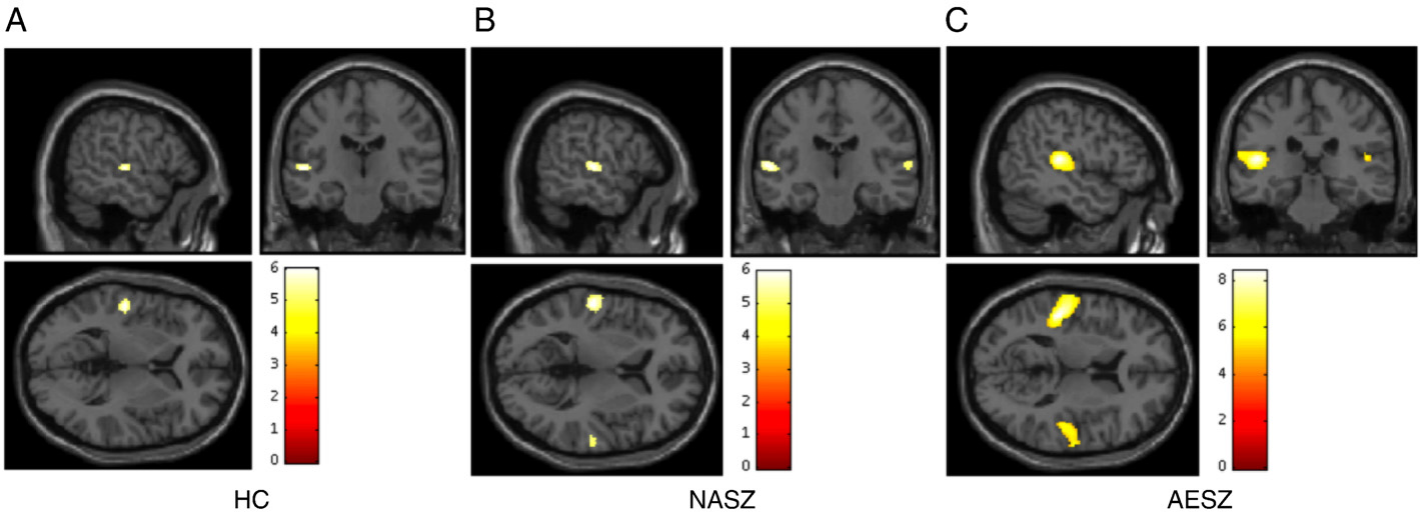
Results of BOLD signals activated by 80-Hz click stimuli compared with resting states. (A) Healthy controls (HC), (peak: left transverse temporal gyrus, [− 54, − 20, 4], *t* = 6.00, *p* < 0.001). (B) Non-acute phase schizophrenia patients (NASZ), (peak: left transverse temporal gyrus/planum temporale, [− 56, − 20, 6], *t* = 5.98, *p* < 0.001) (peak: right superior temporal gyrus, [64, − 22, 8], *t* = 5.18, *p* < 0.001). (C) Acute episode schizophrenia patients (AESZ) (peak: left transverse temporal gyrus/planum temporale, [− 48, − 28, 12], *t* = 8.43, *p* < 0.001) (peak: right planum temporale, [54, − 20, 8], *t* = 6.28, *p* < 0.001). Colored bars represent the *t*-value of the contrast (FWE corrected, *p* < 0.05). The [x, y, z] locations are listed in Montreal Neurological Institute coordinates.

Fig. 3 shows a scattergram of contrast values of ASSR BOLD for each stimulus in the left Brodmann areas 41 and 42. A repeated-measures ANOVA showed no significant main effect of group (F[2, 50] = 2.63, *p* = 0.082), but we found a significant main effect of frequency (F[3,50] = 10.65, *p* < 0.001) and a significant frequency-by-group interaction (F[3,50] = 5.16, *p* < 0.001). Follow up one-way ANOVAs revealed a significant group difference for 80-Hz stimuli (F[2,50] = 8.58, *p* = 0.001), but not 20-Hz (F[2,50] = 2.09, *p* = 0.13), 30-Hz (F[2,50] = 1.02, *p* = 0.37) or 40-Hz stimuli (F[2,50] = 1.08, *p* = 0.35). Post-hoc analyses with Bonferroni corrections revealed the following significant results; AESZ > HC (*p* = 0.001, d = 1.28) and AESZ > NASZ (*p* = 0.018, d = 1.08) in terms of responses to 80-Hz stimuli. These results indicate that the AESZ group showed significantly increased ASSR-BOLD signals in response to 80-Hz stimuli compared with the NASZ and HC groups, while we observed no significant group differences for 20, 30, or 40-Hz stimuli. Fig. 4 shows the BOLD signals activated by 80-Hz click stimuli compared with the resting state in the HC, NASZ, and AESZ groups. Thus, we focused our remaining analyses on a BOLD time course analysis for 80-Hz ASSR stimuli in the left Brodmann areas 41 and 42.

### 3.3 ASSR-BOLD Time Course Analysis in the Left Brodmann Areas 41 and 42

**Fig. 5:**
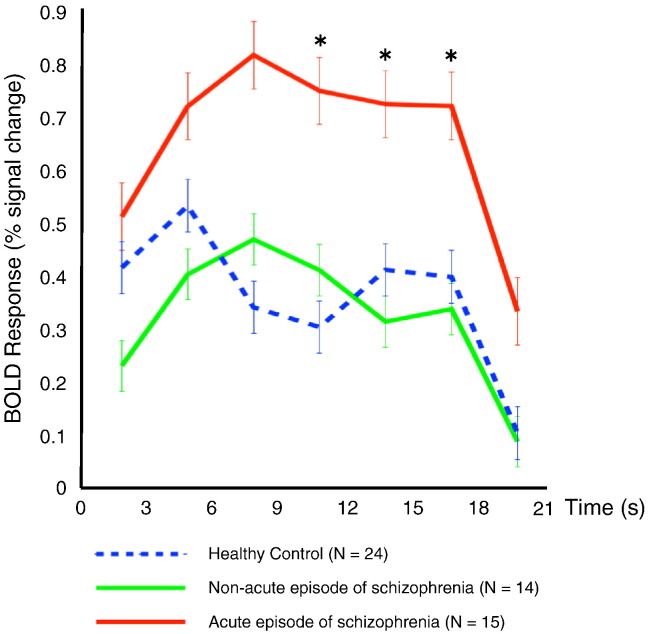
The time course of the 80-Hz ASSR-BOLD percent signal change. The x-axis indicates time (s), and the y-axis indicates BOLD percent signal change. The blue dotted line, green line, and red line indicate the 80-Hz ASSR-BOLD percent signal change in the HC, NASZ, and AESZ groups, respectively. The AESZ group showed a significantly increased BOLD response from 9 to 18 s (*p < 0.05, corrected) compared with both the NASZ and HC groups.

The time course of BOLD percent signal change for 80-Hz ASSR showed different patterns among groups in the left Brodmann areas 41 and 42 (Fig. 5). Quantitatively, a repeated-measures ANOVA revealed a significant main effect of group (F[2,50] = 7.755, *p* = 0.008) and time-by-group interactions (F[14,50] = 4.517, *p* = 0.002). For ANOVAs in each time window, we found significant main effects of group in the 0–3 s (F[2,50] = 3.951, *p* = 0.026), 6–9 s (F[2,50] = 7.007, *p* = 0.002), 9–12 s (F[2,50] = 7.015, *p* = 0.002), 12–15 s (F[2,50] = 6.153, *p* = 0.004), 15–18 s (F[2,50] = 5.042, *p* = 0.01), and 18–21 s (F[2,50] = 3.671, *p* = 0.033) periods. Post hoc tests revealed a significant increase in percent signal change in the 9–18 s period in the AESZ group compared with both the NASZ (9–12 s: *p* = 0.048, d = 0.95; 12–15 s: *p* = 0.006, d = 1.16; 15–18 s: *p* = 0.019, d = 1.12) and HC groups (9–12 s: *p* = 0.002, d = 1.14; 121–15 s: *p* = 0.021, d = 0.92; 15–18 s: *p* = 0.027, d = 0.84).

### 3.4 Correlations between the ASSR and Demographic/Clinical Measurements

**Fig. 6:**
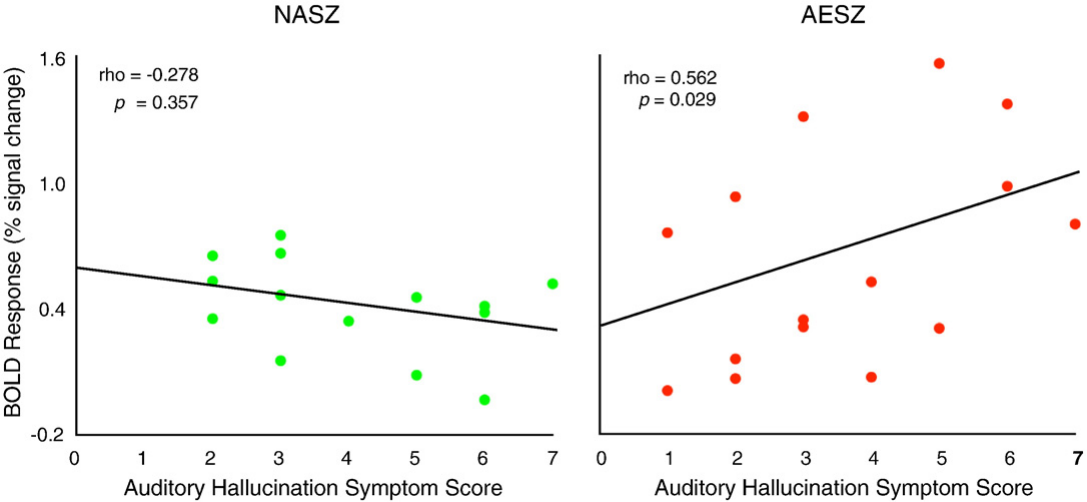
Correlations between auditory hallucination symptom scores and BOLD percent signal change in the left auditory cortex during 80-Hz stimulation in patients with NASZ (left) and AESZ (right). Although we used Spearman's rho to test statistical significance due to the small N, we have also plotted a least squares line for the convenience of the reader.

Because we observed the largest main effect of group in the 9–12 s period, we performed correlational analyses between hallucination scores (0, not present; 7, extremely severe auditory hallucinations) in the PANSS and BOLD percent signal changes in this period. We hypothesized that 80-Hz ASSR-BOLD percent signal changes would be positively correlated with hallucinatory scores. In the AESZ group, we found a significant positive correlation between the BOLD change and auditory hallucinatory experiences (rho = 0.562, *p* = 0.029), but not in the NASZ group (rho = − 0.278, *p* = 0.357) (Fig. 6).

For medication, we found no significant associations between medication dosage and BOLD percent signal change in AESZ (− 0.410 ≤ rho ≤ − 0.058, 0.129 ≤ *p* ≤ 0.839) or NASZ (− 0.231 ≤ rho ≤ 0.240, 0.409 ≤ *p* ≤ 0.899) patients.

## 4 Discussion

In this study, we compared the ASSR-BOLD elicited by 20, 30, 40, and 80-Hz click trains in AESZ, NASZ, and HC groups. AESZ patients showed significantly increased ASSR-BOLD signals to 80-Hz stimuli compared with NASZ and HC in the left auditory cortex, while NASZ patients were not significantly different from HC for any stimuli. These results indicate that schizophrenia patients in the acute state exhibit overactivation of the left auditory cortex during 80-Hz auditory stimulation. With respect to clinical correlations, we found that a higher severity of global auditory hallucinatory experiences was associated with a larger BOLD percent signal change in the left auditory cortex in the AESZ group.

As noted in the introduction section, fMRI can be used to detect both evoked and spontaneous gamma activities during auditory stimulation, and increased spontaneous gamma responses have previously been reported in patients with schizophrenia (Hirano et al., 2015). The present results indicate that schizophrenia patients in the acute state exhibit an overall increase in neural activation in the left auditory cortex during 80-Hz auditory stimulation. Considering the potential association between abnormal spontaneous gamma activities and increased glutamate levels, our data might reflect glutamate toxicity in the left auditory cortex in the acute state of schizophrenia, which may then trigger progressive changes in the left transverse temporal gyrus and left planum temporale.

Accumulating EEG and MEG evidence indicates that patients with schizophrenia show reduced gamma band ASSR in the chronic state (e.g., Kwon et al., 1999, Carlen et al., 2012, O'Donnell et al., 2013) and during the first episode (e.g., Tada et al., 2014). In the present study, however, we found significantly increased ASSR-BOLD signals in response to 80-Hz but not 40-Hz stimuli. Rivolta et al. (2015) reported that resting-state spontaneous neural oscillations in the gamma frequencies were increased by ketamine. NMDAR antagonists increase pyramidal cell activity and extracellular glutamate levels (Homayoun and Moghaddam, 2007), and blocking the NMDAR with ketamine can cause schizophrenia-like symptoms, including cognitive dysfunction, in healthy subjects (Krystal et al., 1994, Fletcher and Honey, 2006). Recently, high gamma (> 60-Hz) band oscillations have become an increasingly frequent subject of interest. For example, Uhlhaas et al. (2011) reported that high gamma band activities may be a fundamental aspect of temporal coding in cortical networks. Although the ASSR-BOLD may not independently reflect cognitive processes, this may be the case for the 80-Hz ASSR, as basic neural circuits that predominantly oscillate at high gamma frequencies might be more strongly implicated in cognitive processes. In another resting-state EEG study, Mitra et al. (2015) reported that patients with schizophrenia at the untreated acute stage showed significantly higher spontaneous activities in high gamma (70 to 100-Hz) band, but not low gamma band. Given the question of why we only found hyperactivation for the 80-Hz stimuli, it is possible that increased spontaneous gamma activities are more apparent for 80-Hz compared with 40-Hz stimuli in the AESZ group. Alternatively, there may be a strong association between BOLD signals and high gamma band electrophysiological activities. The 80-Hz stimulation may be a resonant frequency for ASSR-BOLD responses in the AESZ group, resulting in hyperactivation for the 80-Hz stimuli only.

Given the present data, we are not able to explain why the left auditory cortex was overactivated during 80-Hz ASSR stimulation. However, the left and right transverse temporal gyri and left planum temporale have some differential cytoarchitectonic features (Seldon, 1981, Hutsler and Gazzaniga, 1996) that may be implicated in lateralized activation. Previously, our group reported left-lateralized abnormally-evoked gamma oscillations to vowel sounds in patients with schizophrenia (Hirano et al., 2008). Additionally, Dierks et al. demonstrated increased fMRI activation in the left transverse temporal gyrus during auditory hallucinations in right-handed individuals with schizophrenia (Dierks et al., 1999). In the current study, increased severity of global auditory hallucinatory experiences was associated with larger BOLD percent signal changes in the left auditory cortex in the AESZ group. Thus, the present results may support the view that the left auditory cortex is the site of defective anatomical substrate associated with auditory hallucinations in acute schizophrenia.

We found group differences in the time course of alterations in ASSR-BOLD. Specifically, we found that, unlike the NASZ and HC groups, the AESZ group exhibited a BOLD increase in high gamma-band ASSRs, especially during the period from 9 to 18 s after the onset of 80-Hz stimuli. In the HC group, BOLD signals decreased from 6 to 9 s for the same stimuli. This phenomenon may be associated with ASSR habituation, as demonstrated by an exponential electrophysiological decrease following repeated exposure to stimuli in rats (Prado-Gutierrez et al., 2015). Indeed, AESZ patients may show delayed habituation to 80-Hz stimuli; however, further studies will be needed to clarify this issue.

In interpreting the current study, it is important to consider several possible limitations. First, ASSR-BOLD responses are involved not only in gamma band activities, but broadband activities as well. However, only gamma band oscillations have been highly correlated with hemodynamic responses (Niessing et al., 2005). The 80-Hz stimulation may be a resonant frequency with respect to the activation of ASSR-BOLD signals. Future studies should investigate this using simultaneous EEG-fMRI recordings. Second, our sample size was relatively small (24 HC, 14 NASZ, and 15 AESZ participants), partially due to the challenges of scanning schizophrenia patients in the acute phase. The association between 80-Hz ASSR-BOLD signals and auditory hallucinatory experiences should be confirmed in a larger sample.

In summary, we found that AESZ patients showed significantly increased BOLD signals during an 80-Hz ASSR compared with NASZ and HC participants. This neural overactivation in patients in the acute state of schizophrenia might be related to the pathophysiology of schizophrenia with respect to glutamate toxicity in the left auditory cortex. Thus, the observed increase in BOLD signal patterns during 80-Hz ASSR may represent clinically relevant outcome predictors, leading to new therapeutic options.

## Funding Sources

This work was supported in part by a Grant-in-Aid for Scientific Research (B25293252 to S.K., C23591712 to T.O., B22791129 and 15K09836 to Y.H.), a Grant-in-Aid for Young Scientists (B) (15K19735 to N.O. and 25861044 to H.K.), and the Program for Advancing Strategic International Networks to Accelerate the Circulation of Talented Researchers from the Ministry of Education, Culture, Sports, Science, and Technology, Japan (S2208 to S.K. and T.O.); a Research Grant from the Brain Science Foundation to Y.H.; the Research Group for Schizophrenia to N.O. and H.K.; the Health and Labor Sciences Research Grants for Comprehensive Research on Persons with Disabilities from Japan Agency for Medical Research and Development, AMED (16dk0307047h0002) to S.K.; and JSPS KAKENHI Grant numbers 25117011 and 25117001 to S.K. No additional external funding was received for this study. The funders had no role in study design, data collection, data analysis, interpretation, or writing of this report.

## Conflicts of Interest

The authors declare no conflict of interest.

## Author Contributions

H.K., T.O., Y.H., and T.U. designed the study; H.K., T.O., I.N., and T.U. performed the research; R.K. and S.K. contributed new reagents/analytic tools; H.K., H.M., and T.U. analyzed the data; H.K., T.O., Y.H., and T.U. wrote the paper; H.K. and N.O. recruited participants and acted as clinicians.

## References

[bb0005] Bell M., Milstein R., Beam-Goulet J., Lysaker P., Cicchetti D. (1992). The positive and negative syndrome scale and the brief psychiatric rating scale. Reliability, comparability, and predictive validity. J. Nerv. Ment. Dis..

[bb0010] Carlen M., Meletis K., Siegle J.H. (2012). A critical role for NMDA receptors in parvalbumin interneurons for gamma rhythm induction and behavior. Mol. Psychiatry.

[bb0015] Coyle J.T., Konopaske G. (2016). Glutamatergic dysfunction in schizophrenia evaluated with magnetic resonance spectroscopy. JAMA Psychiatry.

[bb0020] Dale A.M. (1999). Optimal experimental design for event-related fMRI. Hum. Brain Mapp..

[bb0025] Dierks T., Linden D.E., Jandl M. (1999). Activation of Heschl's gyrus during auditory hallucinations. Neuron.

[bb0030] First M., Spitzer R., Gibbon M. (2002). Structure Clinical Interview for DSM-IV-TR Axis I Disorders-Non-patient Edition (SCID-I/NP, 11/2002 Revision).

[bb0035] First M., Spitzer R., Gibbon M., Williams J. (2002). Structured Clinical Interview for DSM-IV-TR Axis I Disorders, Research Version, Patient Edition. (SCID-I/P).

[bb0040] Fletcher P.C., Honey G.D. (2006). Schizophrenia, ketamine and cannabis: evidence of overlapping memory deficits. Trends Cogn. Sci..

[bb0045] Friston K.J., Holmes A.P., Worsley K.J., Poline J.P., Frith C.D., Frackowiak R.S.J. (1994). Statistical parametric maps in functional imaging: a general linear approach. Hum. Brain Mapp..

[bb0050] Goense J.B., Logothetis N.K. (2008). Neurophysiology of the BOLD fMRI signal in awake monkeys. Curr. Biol..

[bb0055] Hatta K., Ito H. (2014). Strategies for early non-response to antipsychotic drugs in the treatment of acute-phase schizophrenia. Clin. Psychopharmacol. Neurosci..

[bb0060] Herdman A.T., Wollbrink A., Chau W., Ishii R., Ross B., Pantev C. (2003). Determination of activation areas in the human auditory cortex by means of synthetic aperture magnetometry. NeuroImage.

[bb0065] Hirano S., Hirano Y., Maekawa T. (2008). Abnormal neural oscillatory activity to speech sounds in schizophrenia: a magnetoencephalography study. J. Neurosci..

[bb0070] Hirano Y., Oribe N., Kanba S., Onitsuka T., Nestor P.G., Spencer K.M. (2015). Spontaneous gamma activity in schizophrenia. JAMA Psychiatry.

[bb0075] Homayoun H., Moghaddam B. (2007). NMDA receptor hypofunction produces opposite effects on prefrontal cortex interneurons and pyramidal neurons. J. Neurosci..

[bb0080] Hutsler J.J., Gazzaniga M.S. (1996). Acetylcholinesterase staining in human auditory and language cortices: regional variation of structural features. Cereb. Cortex.

[bb0085] Kann O., Huchzermeyer C., Kovacs R., Wirtz S., Schuelke M. (2011). Gamma oscillations in the hippocampus require high complex I gene expression and strong functional performance of mitochondria. Brain.

[bb0090] Kay S.R., Fiszbein A., Opler L.A. (1987). The positive and negative syndrome scale (PANSS) for schizophrenia. Schizophr. Bull..

[bb0095] Kayser C., Kim M., Ugurbil K., Kim D.S., Konig P. (2004). A comparison of hemodynamic and neural responses in cat visual cortex using complex stimuli. Cereb. Cortex.

[bb0100] Krystal J.H., Karper L.P., Seibyl J.P. (1994). Subanesthetic effects of the noncompetitive NMDA antagonist, ketamine, in humans. Psychotomimetic, perceptual, cognitive, and neuroendocrine responses. Arch. Gen. Psychiatry.

[bb0105] Kwon J.S., O'Donnell B.F., Wallenstein G.V. (1999). Gamma frequency-range abnormalities to auditory stimulation in schizophrenia. Arch. Gen. Psychiatry.

[bb0110] Li H., Turkoz I., Zhang F. (2016). Efficacy and safety of once-monthly injection of paliperidone palmitate in hospitalized Asian patients with acute exacerbated schizophrenia: an open-label, prospective, noncomparative study. Neuropsychiatr. Dis. Treat..

[bb0115] Logothetis N.K., Augath M., Murayama Y. (2010). The effects of electrical microstimulation on cortical signal propagation. Nat. Neurosci..

[bb0120] Logothetis N.K., Pauls J., Augath M., Trinath T., Oeltermann A. (2001). Neurophysiological investigation of the basis of the fMRI signal. Nature.

[bb0125] Marsman A., van den Heuvel M.P., Klomp D.W., Kahn R.S., Luijten P.R., Hulshoff Pol H.E. (2013). Glutamate in schizophrenia: a focused review and meta-analysis of (1)H-MRS studies. Schizophr. Bull..

[bb0130] Mitra S., Nizamie S.H., Goyal N., Tikka S.K. (2015). Evaluation of resting state gamma power as a response marker in schizophrenia. Psychiatry Clin. Neurosci..

[bb0135] Mouchlianitis E., McCutcheon R., Howes O.D. (2016). Brain-imaging studies of treatment-resistant schizophrenia: a systematic review. Lancet Psychiatry.

[bb0140] Murayama Y., Biessmann F., Meinecke F.C. (2010). Relationship between neural and hemodynamic signals during spontaneous activity studied with temporal kernel CCA. Magn. Reson. Imaging.

[bb0145] Niessing J., Ebisch B., Schmidt K.E., Niessing M., Singer W., Galuske R.A. (2005). Hemodynamic signals correlate tightly with synchronized gamma oscillations. Science.

[bb0150] O'Donnell B.F., Vohs J.L., Krishnan G.P., Rass O., Hetrick W.P., Morzorati S.L. (2013). The auditory steady-state response (ASSR): a translational biomarker for schizophrenia. Suppl. Clin. Neurophysiol..

[bb0155] Olabi B., Ellison-Wright I., McIntosh A.M., Wood S.J., Bullmore E., Lawrie S.M. (2011). Are there progressive brain changes in schizophrenia? A meta-analysis of structural magnetic resonance imaging studies. Biol. Psychiatry.

[bb0160] Oldfield R.C. (1971). The assessment and analysis of handedness: the Edinburgh inventory. Neuropsychologia.

[bb0165] Ollinger J.M., Corbetta M., Shulman G.L. (2001). Separating processes within a trial in event-related functional MRI. NeuroImage.

[bb0170] Owen M.J., Sawa A., Mortensen P.B. (2016). Schizophrenia. Lancet.

[bb0175] Prado-Gutierrez P., Castro-Farinas A., Morgado-Rodriguez L., Velarde-Reyes E., Martinez A.D., Martinez-Montes E. (2015). Habituation of auditory steady state responses evoked by amplitude-modulated acoustic signals in rats. Audiol Res..

[bb0180] Rivolta D., Heidegger T., Scheller B. (2015). Ketamine dysregulates the amplitude and connectivity of high-frequency oscillations in cortical-subcortical networks in humans: evidence from resting-state magnetoencephalography-recordings. Schizophr. Bull..

[bb0185] Ross B., Herdman A.T., Pantev C. (2005). Right hemispheric laterality of human 40 Hz auditory steady-state responses. Cereb. Cortex.

[bb0190] Salomon J.A., Vos T., Hogan D.R. (2012). Common values in assessing health outcomes from disease and injury: disability weights measurement study for the Global Burden of Disease Study 2010. Lancet.

[bb0195] Scheeringa R., Fries P., Petersson K.M. (2011). Neuronal dynamics underlying high- and low-frequency EEG oscillations contribute independently to the human BOLD signal. Neuron.

[bb0200] Scholvinck M.L., Maier A., Ye F.Q., Duyn J.H., Leopold D.A. (2010). Neural basis of global resting-state fMRI activity. Proc. Natl. Acad. Sci. U. S. A..

[bb0205] Seldon H.L. (1981). Structure of human auditory cortex. I. Cytoarchitectonics and dendritic distributions. Brain Res..

[bb0210] Sivarao D.V. (2015). The 40-Hz auditory steady-state response: a selective biomarker for cortical NMDA function. Ann. N. Y. Acad. Sci..

[bb0215] Tada M., Nagai T., Kirihara K. (2014). Differential alterations of auditory gamma oscillatory responses between pre-onset high-risk individuals and first-episode schizophrenia. Cereb. Cortex.

[bb0220] Takahashi T., Suzuki M., Zhou S.Y. (2010). A follow-up MRI study of the superior temporal subregions in schizotypal disorder and first-episode schizophrenia. Schizophr. Res..

[bb0225] Tsuchimoto R., Kanba S., Hirano S. (2011). Reduced high and low frequency gamma synchronization in patients with chronic schizophrenia. Schizophr. Res..

[bb0230] Uhlhaas P.J., Pipa G., Neuenschwander S., Wibral M., Singer W. (2011). A new look at gamma? High- (> 60 Hz) gamma-band activity in cortical networks: function, mechanisms and impairment. Prog. Biophys. Mol. Biol..

[bb0235] Uhlhaas P.J., Roux F., Rodriguez E., Rotarska-Jagiela A., Singer W. (2010). Neural synchrony and the development of cortical networks. Trends Cogn. Sci. (Regul. Ed.).

