# Corrigendum to “Increased BOLD Signals Elicited by High Gamma Auditory Stimulation of the Left Auditory Cortex in Acute State Schizophrenia” [EBioMedicine 12 (2016) 143-149]

**DOI:** 10.1016/j.ebiom.2017.01.002

**Published:** 2017-01-06

**Authors:** Hironori Kuga, Toshiaki Onitsuka, Yoji Hirano, Itta Nakamura, Naoya Oribe, Hiroaki Mizuhara, Ryota Kanai, Shigenobu Kanba, Takefumi Ueno

**Affiliations:** a Department of Neuropsychiatry, Graduate School of Medical Sciences, Kyushu University, 3-1-1 Maidashi, Higashiku, Fukuoka 812-8582, Japan; b Division of Clinical Research, National Hospital Organization, Hizen Psychiatric Center, 160 Mitsu, Yoshinogari-cho, Kanzaki-gun, Saga 842-0192, Japan; c Graduate School of Informatics, Kyoto University, 36-1 Yoshida-Honmachi, Sakyo-ku, Kyoto 606-8501, Japan; d Araya Brain Imaging, 1-6-15-301, Hirakawa-cho, Chiyoda-ku, Tokyo 102-0093, Japan

The authors wish to point out that there are some errors in the Result section of the article, whereby freedoms should have been corrected below.

3.2.1 Main Effect of Group and Stimulation

The freedoms should be expressed as F [3, 200], and not F [3200]. The correct text should read as follows:

Our full-factorial ANOVA revealed no significant group differences. The main effect of frequency was associated with significant bilateral activity in Brodmann areas 41 and 42 (left: − 54, − 26, 6, cluster size = 656, F [3, 200] = 15.38, FWE corrected *p* < 0.001; right: 58, − 20, 4, cluster size = 297, F [3, 200] = 9.32, FWE corrected *p* = 0.003), indicating that the 80-Hz stimulation evoked significantly larger BOLD responses, compared with the other stimulation frequencies.

3.2.2 ASSR-BOLD contrast in Brodmann areas 41 and 42

The freedom of main effect of frequency should be expressed as F [3, 48], and not F [3, 50]. The freedom of frequency-by-group interaction should be expressed as F [6, 98], and not F [3, 50]. The correct text should read as follows:

Fig. 3 shows a scattargram of contrast values of ASSR BOLD for each stimulus in the left Brodmann areas 41 and 42. A repeated-measures ANOVA showed no significant main effect of group (F[2, 50] = 2.63, *p* = 0.082), but we found a significant main effect of frequency (F[3, 48] = 10.65, *p* < 0.001) and a significant frequency-by-group interaction (F[6, 98] = 5.16, *p* < 0.001).

3.3. ASSR-BOLD Time Course Analysis in the Left Brodmann areas 41 and 42

The freedom of time-by-group interaction should be expressed as F [12, 92], and not F [14, 50]. The correct text should read as follows:

The time course of BOLD percent signal change for 80-Hz ASSR showed different patterns among groups in the left Brodmann areas 41 and 42 (Fig. 5). Quantitatively, a repeated-measures ANOVA revealed a significant main effect of group (F[2,50] = 7.755, *p* = 0.008) and time-by-group interactions (F[12,92] = 4.517, *p* = 0.002).

The authors also wish to point out that the word ‘neural over activation’ in the Abstract of this article should be corrected to ‘neural over-activation’, as in Keywords section of the article information.

These errors do not change the scientific conclusions of the article in any way.

